# Chromosomal disruption and rearrangements during murine sarcoma development converge to stable karyotypic formation kept by telomerase overexpression

**DOI:** 10.1186/s12929-016-0230-y

**Published:** 2016-02-03

**Authors:** Robson José de Oliveira-Júnior, Carlos Ueira-Vieira, Angela Aparecida Servino Sena, Carolina Fernandes Reis, José Roberto Mineo, Luiz Ricardo Goulart, Sandra Morelli

**Affiliations:** Institute of Genetics and Biochemistry, Federal University of Uberlândia, Uberlândia, MG Brazil; Institute of Biomedical Sciences, Federal University of Uberlândia, Uberlândia, MG Brazil; Department of Medical Microbiology and Immunology, University of California Davis, Davis, CA USA

**Keywords:** Carcinogenesis, Chromosome instability, Cancer, Telomerase

## Abstract

**Background:**

Tumor initiation presents a complex and unstable genomic landscape; one of the earliest hallmark events of cancer, and its progression is probably based on selection mechanisms under specific environments that lead to functional tumor cell speciation. We hypothesized that viable tumor phenotypes possess common and highly stable karyotypes and their proliferation is facilitated by an attuned high telomerase activity. Very few investigations have focused on the evolution of common chromosomal rearrangements associated to molecular events that result in functional phenotypes during tumor development.

**Results:**

We have used cytogenetic, flow cytometry and cell culture tools to investigate chromosomal rearrangements and clonality during cancer development using the murine sarcoma TG180 model, and also molecular biology techniques to establish a correlation between chromosome instability and telomerase activity, since telomeres are highly affected during cancer evolution. Cytogenetic analysis showed a near-tetraploid karyotype originated by endoreduplication. Chromosomal rearrangements were random events in response to in vitro conditions, but a stable karyotypic equilibrium was achieved during tumor progression in different in vivo conditions, suggesting that a specific microenvironment may stabilize the chromosomal number and architecture. Specific chromosome aberrations (marker chromosomes) and activated regions (rDNAs) were ubiquitous in the karyotype, suggesting that the conservation of these patterns may be advantageous for tumor progression. High telomerase expression was also correlated with the chromosomal rearrangements stabilization.

**Conclusions:**

Our data reinforce the notion that the sarcoma cell evolution converges from a highly unstable karyotype to relatively stable and functional chromosome rearrangements, which are further enabled by telomerase overexpression.

## Background

The hallmarks of cancer were recently revisited, and included Deregulating cellular energetics and avoiding immune destruction as an emerging characteristic, and tumor-promoting inflammation, genome instability and mutations as enabling characteristics. Multiple genomic mutations are considered responsible for the malignant transformation of normal cells, which includes: capacity of tissue invasion and metastasis, insensitivity to antigrowth signals, sustained angiogenesis, ability to evade apoptosis, self-sufficient growth signals, limitless replication potential [1] evasion of immune surveillance [2], DNA damage and several causative conditions of cellular stress such as DNA replication, mitosis and oxidative proteotoxic and metabolic processes [3]. However, an interesting emphasis has been given to the genomic instability as one of the most important hallmarks because of its presence in all cancer stages [4]. The final stage of malignant cells is determined by multiple mutations that confer distinct fitness advantages, and occur stochastically. The characteristic of each tumor depends on the complement of mutations acquired by its cells [5].

There are two conflicting views on carcinogenesis; “genocentric view” proposes that favorable gene mutations and epigenetic alterations are early events in cancer, leading to altered cell phenotypes and clonal expansion [6, 7]. A second view is explained by the chromosomal theory of cancer based on aneuploidy [8], which is considered a solid cancer hallmark [9]. Aneuploidy may be required for tumor establishment in mice, and it may work in conjunction with intragenic mutations during tumorigenesis [10]. This is corroborated by observations that some genetic alterations associated with tumor initiation or proliferation events can be mediated by large chromosomal changes [11]. The presence or absence of specific chromosomes from the chromosome set, the increasing number of chromosome copies or the presence of some marker chromosomes can determine whether a cell line is more or less invasive, thereby directing the type of treatment to be adopted. Even cells with similar ploidy may show specific rearrangements that increase their metastatic ability [12].

Telomerase expression has also an important role in tumor growth and cell immortalization, and its overexpression may be associated with chromosomal rearrangements maintenanceand increases tolerance to chromosomal instability. Its reactivation is a critical event that promotes the tumor proliferation by removing the barrier of telomeric shortening [13, 14], once that telomeric maintenance is essential for the cell immortalization [15].

We have performed a detailed analysis of chromosomal rearrangements during tumor development of the murine cell line derived from sarcoma 180 (TG180) under in vivo and in vitro conditions, and demonstrated that equilibrium of the chromosomal architecture could be established in vivo as opposed to in cell culture conditions, where a remarkable chromosome instability is observed. Furthermore, the in vivo karyotypic stabilization was followed by an increase in the telomerase activity. Our results corroborate the chromosomal theory of cancer, by evidencing that viable tumor phenotypes possess common and highly stable karyotypes and their proliferation is facilitated by telomerase overexpression.

## Methods

### Animals, cells and culture conditions

TG180 cells were purchased from American Type Culture Collection (ATCC, Manassas, USA) and grown in vitro using RPMI-1640 medium, with 10 % fetal calf serum (FCS), 25 mM HEPES, 1 % penicillin-streptomycin, and 2 mM L-glutamine. The in vivo maintenance of the cells was done by the inoculation of 300 μL of cells (1.0 × 107 cells cells) in the peritoneum of three Balb-c male mice, weighting ± 20 g. Animals were kept in the Animal Experimentation Laboratory (LEA) of the Federal University of Uberlândia under controlled conditions. Animals were housed under standard conditions (22 ± 1 °C, humidity 60 ± 5 %, 12 h light/12 h dark cycle) with food and water ad libitum. All procedures for the handling, use and euthanasia of these animals followed the rules of the Brazilian Society for Laboratory Animals Science, and was approved by the Ethics Committee in Animal Research of the Federal University of Uberlândia, Brazil (CEUA/UFU N. 039/09) and every effort was made to minimize suffering.

### Cell line cytogenetic characterization

Karyotypic analysis of cells was conducted in the Animal Cytogenetic Laboratory of the Federal University of Uberlândia. The mitotic chromosomes were obtained using the method described elsewhere [16]. The constitutive heterochromatin was revealed using the C-Band [17] and the staining with the fluorochromes chromomycin A3 and Hoechst 33258 [18]. Chromosomes banding patterns were obtained by C- and G-banding [19], and by *Dde*I- and *Bam*HI-restriction enzyme digestions [20]. The Nucleolus Organizing Regions (NORs) were detected by the Ag-NOR impregnation [21]. Chromosomes characterizations were performed as described elsewhere [22] through conventional optical microscopes, epifluorescence microscope and AMG EVOS® fl Digital Inverted Fluorescence Microscope.

### Ploidy analysis by flow cytometry

TG180 cells were collected and washed twice with PBS followed by fixation in 1 % formaldehyde for 1 h and permeabilization with 70 % ethanol overnight at 4 °C. Cells were spun down, resuspended and incubated in 1 mL solution containing 40 μg/mL of propidium iodide (PI) and 100 mg/mL RNase A at 37 °C in the dark for 30 min. Under these staining conditions, signal due to residual double-stranded RNA is negligible and relative intensity of red fluorescence corresponds to DNA content [23]. Chicken erythroid nuclei were used as reference cells to determine the position of the diploid peak (2n). Cell fluorescence intensity and size were measured using AccuriC6 flow cytometer (BD Biosciences, San Diego, CA). Data were analyzed using FlowJo 7.6.1 (Tree Star Inc, Ashland, OR). The established criterion for ploidy of TG180 cells was based on 2n control cells peak/plot, subsequently the horizontal right displacement in the graphs represented a proportional increase of ploidy.

### Clonogenic assay

To perform clonal expansion the viability of cells grown in bottles of 25 cm^2^ was verified by the trypan blue exclusion test. Subsequently these cells were resuspended in medium and diluted to a ratio of 1 cell/μL. An aliquot of 1 μL per well was transferred to 96-well culture microplates containing 200 μL of complete medium in each well and examined under an inverted microscope (Olympus). The wells with only one cell were identified and cell growth was monitored to obtain an adequate number of cells for chromosomal analysis and for inoculation of these tumor cells into mice.

### Total RNA extraction and reverse transcription

Total RNA was extracted from TG180 and normal mice cells using the Trizol reagent according to the manufacturer’s instructions (Invitrogen, Inc.). Reverse transcription (RT) was accomplished by adding 1 μg of total RNA from each sample to a final volume of 20 μL (completed with diethylpyrocarbonate (DEPC) treated water) containing 10 units of RNase inhibitor, 40 units of MMLV reverse transcriptase (RT), 1x MMLV-RT buffer, 200 μM of each dNTP and 6 μM of random hexamer primers and the solution incubated at 37 °C for 1 h and then 95 °C for 5 min.

### RT-PCR of the samples

The cDNA was co-amplified in the same PCR reaction for the target (*M*-*Tert*) and control (*actin*) genes. For the *M-Tert* gene (accession number NM_009354.1) the primers were: sense 5′-GGATTGCCACTGGCTCCG-3′; antisense 5′-TGCCTGACCTCCTCTTGTGAC-3′. The *actin* constitutive gene (accession number NM_007393.2) was used as an internal positive control to normalize the products of the amplification reactions, and the primers were: sense 5′-GGCACCACACCTTCTACAATG-3′ e antisense 5′-GTGGTGGTGAAGCTGTAG-3′. Primers were designed for selective amplification of RNA, in which both primer ends (5′ and 3′) belonged to two adjacent exons. To check for genomic DNA contamination PCR reactions were also performed using total RNA as template, but no amplification was observed, demonstrating that all samples had no contaminant genomic DNA.

Amplification was carried out by adding 2 μL of primary cDNA to a 25 μL PCR mixture consisting of 200 μM of each dNTP, 0.4 μM of the primer pair for *M-Tert* or *Actin*, 2.0 mM MgCl_2_, 1.5 unit of Taq DNA polymerase and 1x buffer. The reactions were incubated at 95 °C for 3 min, followed by 35 (*M*-*Tert*) or 27 (*Actin*) cycles at 95 °C for 30 s, 59 °C (*M*-*Tert*) or 55 °C (*Actin*) for 40 s and 72 °C for 40 s, with a final extension of 10 min at 72 °C. The ideal number of PCR cycles (35 and 27) was determined when the co-amplification of both genes reached the exponential phase.

### Relative levels of gene expression

The *M-Tert* and *Actin* gene amplicons obtained were analyzed and quantified based on the staining intensities of the corresponding bands as assessed using the ImageMaster VDS software program, version 2.0 (Amersham Biosciences). The relative levels of *M-Tert* were obtained for each sample by normalizing the densitometric readings using the ratio M-*Tert*/*Actin*.

### RNA preparation, cDNA synthesis and quantitative real-time RT-PCR

RNA was extracted from tumor tissues using the Trizol reagent (Invitrogen) and RNA mass was determined on a NanoDrop™ 1000 spectrophotometer (Thermo Scientific). cDNA synthesis was carried out using SuperScript II First-Strand Synthesis System for RT-PCR using oligo (dT) primer (Invitrogen) using 1 μg of total RNA. The RNA was extracted from TG180 murine cell line, murine whole blood and NIH-3 T3 murine cell line. The amplification of fragments corresponding to each gene was performed using the following primers: telomerase-F: 5′-TGGCTTGCTGCTGGACACTC-3′ and telomerase-R: 5′-TGAGGCTCGTCTTAATTGAGGTCTG-3′; GAPDH was amplified with primers: 5′-GCACAGTCAAGGCCGAGAAT-3′ (forward) and 5′-GCCTTCTCCATGGTGGTGAA-3′ (reverse) and served as an internal control to normalize expression data and to verify integrity of the cDNA. In order to evaluate similar PCR amplification efficiencies of target genes and GAPDH genes, a serial dilution analysis was performed using cDNA synthesized from total RNA from normal and tumor cell lines.

All quantitative real time PCR (qRT-PCR) reactions were conducted using the SYBR Green detection reagent (Applied Biosystems). Conditions for PCR amplication of target genes were: 50 °C for 2 min and 94 °C for 5 min, followed by 10 cycles of 94 °C for 30s, primer annealing 57 °C for 2 min and 72 °C for 90s. At the end of each cycle the temperature decreased 0.5 °C, followed by 30 cycles of 94 °C for 30 s, 50 °C for 30 s and 72 °C for 90 s, ending the reaction with 72 °C for 15 min. A melting curve analysis was generated to determine amplification efficiency and specificity (60–90 °C with a heating rate of 0.2 °C/s and continuous fluorescence measurement). Product purity, size and absence of primer dimmers were confirmed by the DNA melting curve analysis and by agarose gel electrophoresis. Relative gene expression of the target gene was calculated by using the ΔΔCT method. GAPDH amplification was used as normalization control for evaluation of gene expression levels.

### Statistical analysis

The statistical analysis concerning the distribution of chromosome numbers was performed by using a confidence interval for proportions by Student *t* test. The graphics and the statistical analysis for the telomerase expression were performed in the Statview for Windows version 4.57 (Abacus Concepts, Inc., Copyright 1992–1996). *P* values <0.05 were considered significant. Statistical analyses of real time PCR were conducted by the statistical program GraphPad Prism 6 for windows, version 6.01. Comparisons among groups of data were made using Two-way analysis of variance (ANOVA), followed by the Bonferroni posttest. *P* value <0.05 was considered statistically significant. All results were presented as mean ± SD.

## Results

### Classic cytogenetic analysis reveals cell heterogeneity, near-tetraploidy and conservation of specific chromosomes

**Fig. 1:**
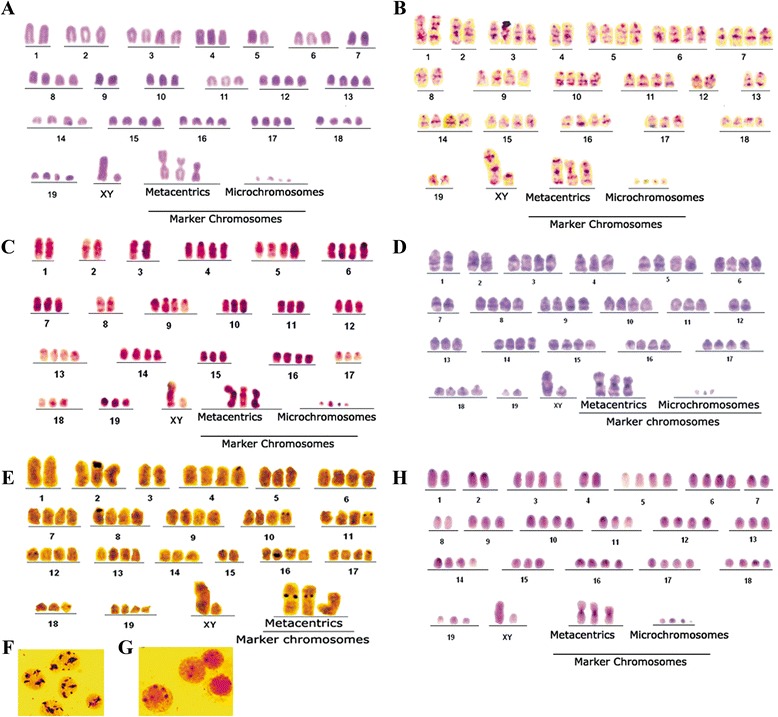
TG180 representative karyotypes under different staining and banding techniques. **a** conventional Giemsa staining. **b** Restriction enzyme *Dde* I. **c** Restriction enzyme *Bam* HI. **d** G-Banding. **e** Silver nitrate impregnation, evidencing Nucleolus Organizer Regions (darker regions on chromosomes). **f** TG180 nuclei impregnated with silver nitrate, evidencing the nucleoli (darker regions on nuclei). Note the greater amount and disorganization of tumor cells nucleoli. **g** Nuclei of mice bone marrow cells (normal cells). Note the presence of a constant organization. **h** C-Banding

Chromosome counting revealed that TG180 is a heterogeneous cell line, with chromosome number ranging from 16 to 142 in the ascetic tumor, with a modal number of 68 chromosomes. Conventional Giemsa karyotype suggested a near-tetraploid complement and revealed the constant presence of tree metacentric and four micro-chromosomes that were considered markers of the cell line (Fig. 1a). Restriction enzyme banding with DdeI and BamHI, and G-banding (Fig. 1b, c and d, respectively) produced specific transversal banding patterns, which allowed the determination of the appropriate chromosome pairing and karyotype assembly. Tetrasomy was frequently observed in several chromosomes, confirming the near-tetraploid complement. Metacentric chromosomes were strictly observed in single copies, suggesting that its origin occurred after polyploidization.

**Fig. 2:**
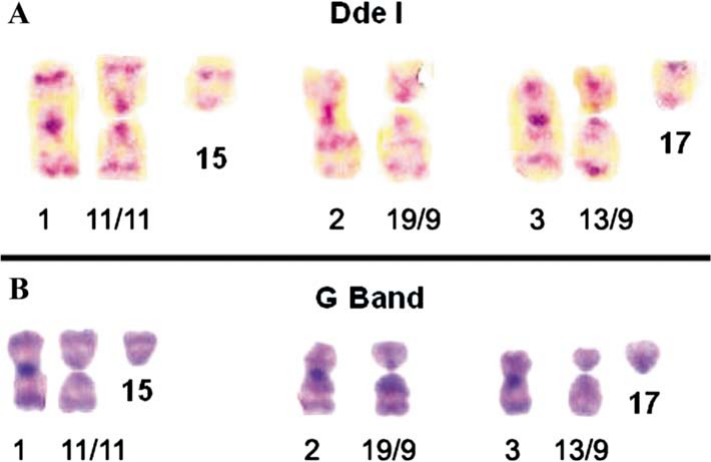
Translocations involved in origin of metacentric marker chromosomes of TG180 cell line. **a** TG180 chromosomes treated with *Dde* I. **b** TG180 chromosomes submitted to G-banding

Analyzing the banding pattern generated by the restriction enzyme Dde I (Fig. 2a) and G-Banding (Fig. 2b), we propose that the largest metacentric chromosome was originated from the fusion of two chromosomes 11 or between one chromosome 11 and one 15. The second metacentric chromosome may be a result of the translocation between the chromosome 19 and 9, while the third metacentric chromosome could be derived from the union of the chromosomes 13 and 19 or 13 and 17.

The metaphases of normal cells from mice were impregnated with silver nitrate and NORs were shown on one chromosome of six different homologous pairs (12, 15, 16, 17, 18 and 19). However, in TG180 cell metaphases at least 11 chromosomes with active NORs were observed (Fig. 1e). In addition to the six chromosome pairs with active NORs observed in normal cells, TG180 cells also presented them on chromosomes 2 (with a large telomeric amplification), 4, 8, 10, 11 and in the centromeric region of two metacentric markers. The TG180 cells presented highly active nucleoli (Fig. 1f) that were markedly disorganized and abundant when compared to normal cells (Fig. 1g).

**Fig. 3:**
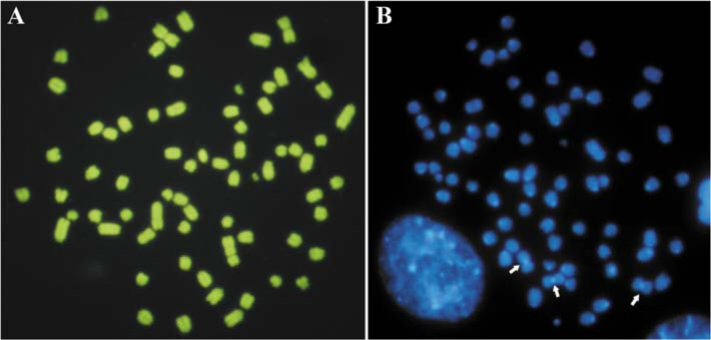
Photomicrographs of TG180 chromosomes stained with different fluorochromes. **a** TG180 metaphase stained with Chromomycin A_3_, note that there are no fluorescent blocks, indicating that heterochromatin is not “rich” in G-C bases. **b** TG180 metaphase stained with Hoechst 33258. Arrows indicate fluorescent heterochromatin blocks “rich” in A-T bases

Constitutive heterochromatin blocks (C-band) were shown to be pericentromeric in most chromosomes, except in metacentric markers, which showed large centromeric blocks (Fig. 1h). Concerning to heterochromatin composition, no GC-rich island was evidenced using CMA3, even when counter-stained with Distamycin A (Fig. 3a). These heterochromatic blocks were AT-rich, since they were positively stained with Hoechst 33258 (Fig. 3b).

**Fig. 4:**
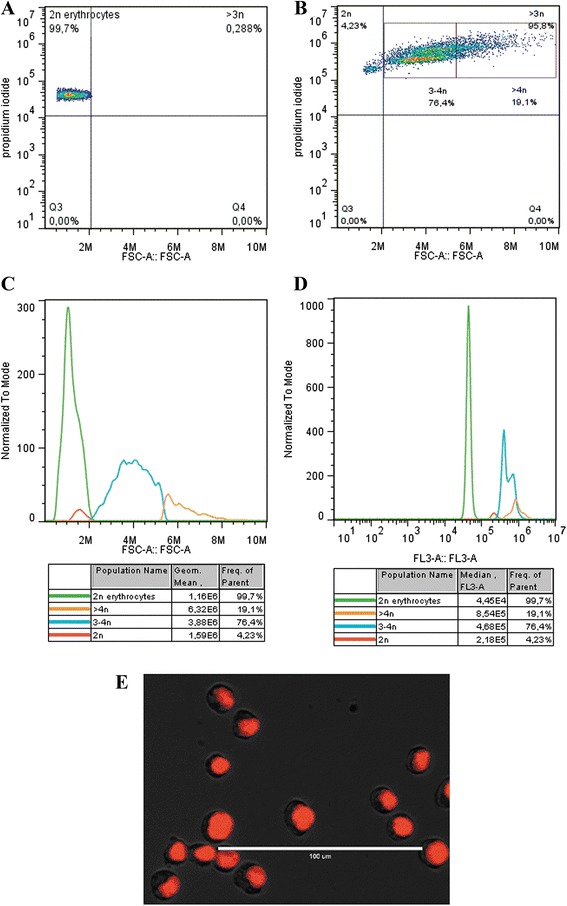
Analysis of TG180 cell population by flow cytometry. **a** and **b** Dot plots showing the ploidy distribution of TG180 cell population compared to the pattern of 2n chicken erythrocytes, respectively. Histogram of TG180 cell population subdivided according to its size in (**c**), and to its DNA amount (**d**), using PI staining. **e** Fluorescence micrograph of TG180 cells evidencing different nucleus size (*red color*) using PI staining

As evidenced by cytogenetic analysis, flow cytometry also revealed a heterogeneous cell line, with a higher amount of cells with near-tetraploid characteristics. Dot-plots revealed that TG180 cells exhibited a remarkable shift in DNA content compared to 2n reference cells (chicken erythrocytes) (Fig. 4a and b). Also, the histograms showed wider distribution of nuclear sizes (Fig. 3c) and broader range of DNA contents (Fig. 4d) in the TG180 cells, represented by 76.4 % of cells ranging 3–4n, which is consistent with the variation of chromosomal numbers and aneuploidy detected in cytogenetic analyzes. A fluorescence image displays the propidium iodide-stained nuclei characterized by different sizes (Fig. 4e).

### Overexpression of telomerase in TG180

**Fig. 5:**
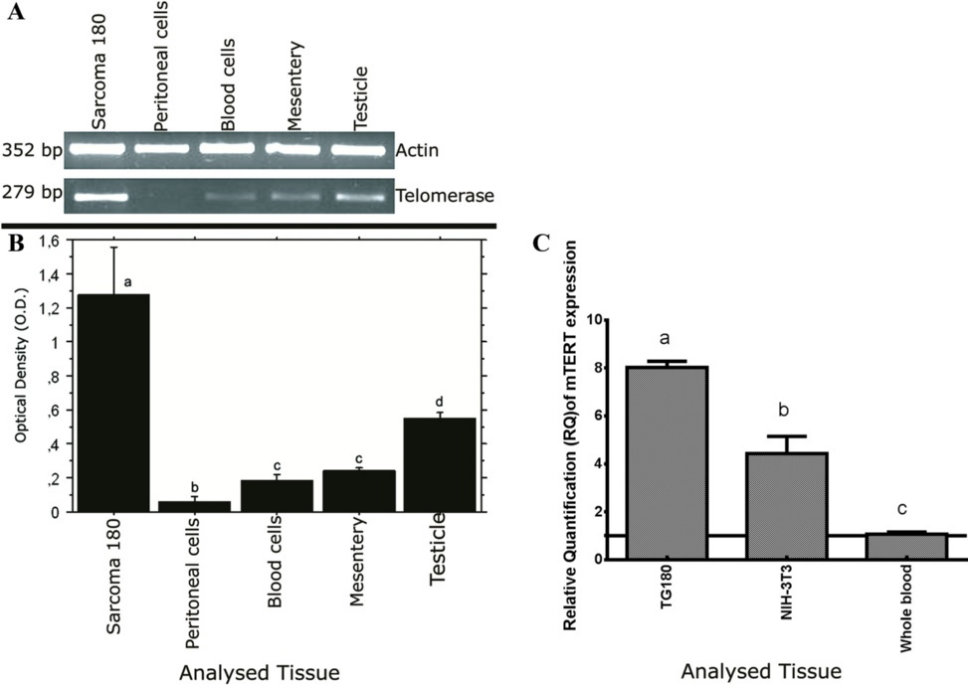
Analysis of telomerase expression by RT-PCR and Real-Time PCR. **a** Agarose gel of RT-PCR products of the genes telomerase and actin of TG180 and normal tissues of mice. **b** Representative graphic of the RT-PCR semi-quantitative analysis of telomerase expression. The results represent the mean ± standard deviation of three independent experiments. It was used the t-student test and *p* values <0.05 were considered statistically significant. Bars with the same letter have no statistical difference. **c** Graphic representation of the relative quantification of expression levels of mouse telomerase gene (mTERT). Columns with the same letter did not show statistical difference. *P* value < 0,05

Semi-quantitative expression of the telomerase (RT-PCR) was analyzed in TG180 cells, and expression was compared among four normal mice tissues (peritoneal cells, blood, testicle and mesentery) as controls, which were normalized with actin gene expression. The telomerase expression in TG180 cells was much higher than those found in normal tissues with the same embryonic origin of the cell line as peritoneal cells, blood and mesentery. Even when compared to testicle, a tissue with intense proliferative activity, TG180 cell line presented *M-Tert* RNA levels at least twice higher (Fig. 5a and b). Although this cell line was composed by a heterogeneous population, it is probable that the telomerase overexpression was primarily produced by 68-chromosome cells, which were the most prevalent (~80 %) cell sub-population.

Comparing telomerase (mTert) expression levels of TG180 sarcoma, whole Blood and NIH-3 T3 cell line using real time PCR (Fig. 5c), it was possible to verify that TG180 sarcoma cell line showed the highest levels of expression of this enzyme. RNA levels of TG180 were almost twice higher than NIH-3 T3 cell line and almost eight times higher than whole blood cells. Comparing the results of TG180 sarcoma and whole blood (mTert) expression levels using real time PCR with semiquantitative RT-PCR, it is possible to verify that these methods obtained almost identical results, indicating that telomerase is really overexpressed in TG180 cells in relation to the other cells analyzed.

### In vitro, but not in vivo, microenvironment induces chromosomal alterations during cell line maintenance

**Fig. 6:**
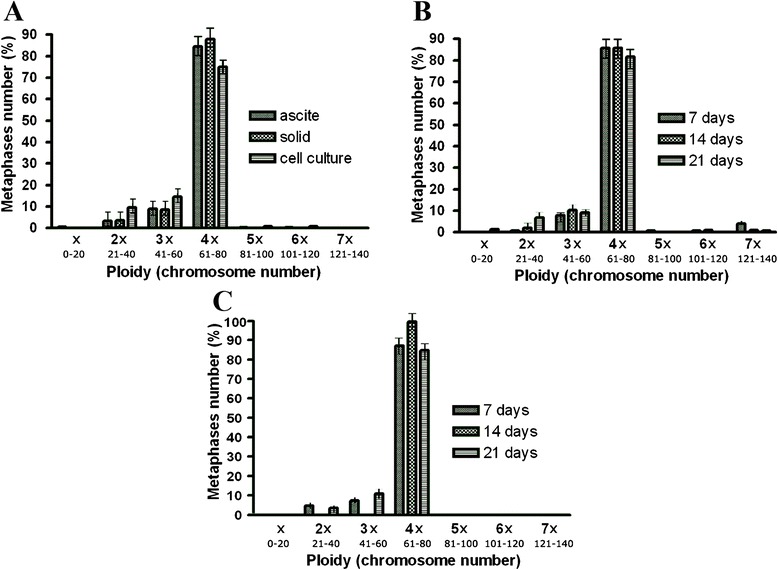
Chromosome number distribution of TG180 according to types of cell line maintenance and tumor progression time. **a** type of cell line maintenance. **b** Progression Time of ascites tumor. **c** Progression time of solid tumor. Confidence interval for proportions by t-student test at 0.05 significance level. Bars represent ± SEM

In order to verify the microenvironment influence in the chromosome balance, chromosome counting was performed on three different types of cellular maintenance: intraperitoneal inoculation (ascitic tumor), intramuscular inoculation (solid tumor) and cell culture (Fig. 6a). For in vivo maintenance, three stages of tumor progression (7 days, 14 days and 21 days of tumor development) have been compared in ascites (Fig. 6b) and solid tumors (Fig. 6c). It was observed the same pattern of chromosome distribution in both cases of in vivo maintenance (ascite and solid tumor) and the different times of tumor progression (days of tumor development) also did not influenced ploidy distribution of tumor cells. A total of 1050 metaphases were analyzed and most of them (over 80 %) showed a near-tetraploid chromosome complement (modal number of 68 chromosomes) in all stages of tumor progression in ascitic and solid tumor.

**Fig. 7:**
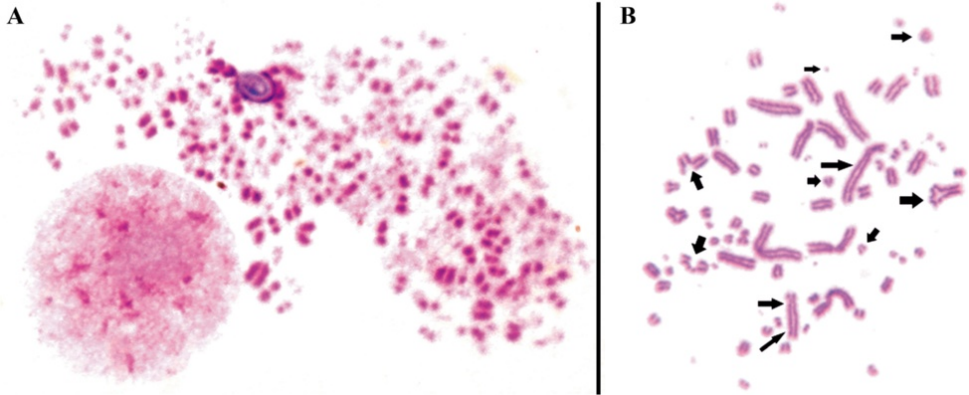
Giemsa stained metaphases of TG180 kept in culture. **a** chromosome pulverization. **b** Metaphase showing diverse chromosome aberrations such as chromatid breaks, chromosome fragmentation and microchromosomes, rings, dicentric chromosomes, triradials and chromosome amplification. Black arrows indicate chromosomal aberrations

In contrast, despite the higher percentage of tetraploid cells and absence of statistically significant difference according to t-student test, the in vitro maintenance showed a tendency to have a wider distribution of ploidy, with a discrete increment in the number of diploid and triploid cells, when compared with in vivo tumor maintenance. It was also observed an increased number of chromosomal aberrations in cells under in vitro maintenance, with several chromosome breaks, chromosome associations and even chromosome pulverization (Fig. 7a and b). This trend to have a wider ploidy distribution was confirmed in the next experiments (clonogenic assay), in which tumor cells passed through long time culture. These results show that in vivo environment tend to select more stable cytotypes, which are adapted to live in this situation, in this case the near-tetraploid ones. The in vitro environment, due to its less controlled conditions, induce the cells to generate a wider variety of cytotypes, in order to ensure that one of these genotypes is effective in survive under such adverse conditions.

**Fig. 8:**
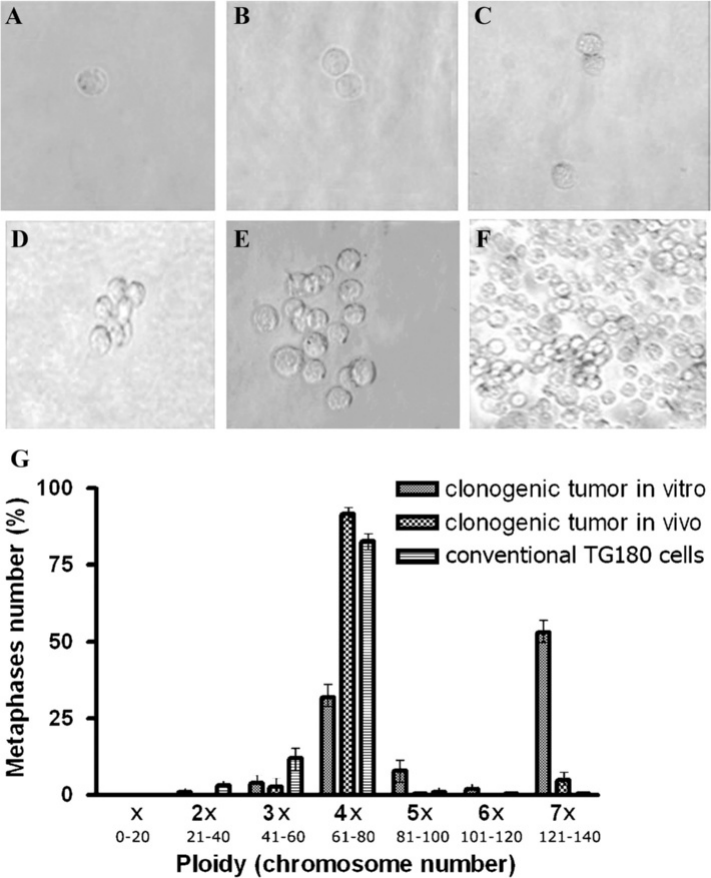
Photomicrographs and Chromosome number distribution of TG180 cells submitted to clonogenic assay. **a**–**f** Different stages of the clonogenic assay, in which a cell was individualized (A) and its development was monitored on an inverted microscope. **g** Representative graphic of chromosome number distribution from TG180 cells obtained by clonal expansion kept in vitro and in vivo in comparison with conventional TG180 cell line. 400 X magnification

Seeking to analyze the chromosome inheritance pattern of TG180, a clonogenic assay was performed. Single cells were distributed in 50 wells of a cell culture microplate. Only one out of these 50 cells showed clonogenic capacity. Development was monitored and photographed (Fig. 8a–f). When culture reached an appropriated number of cells (after a month of culture maintenance), an amount of the cell culture was used to perform cytogenetic analysis, which revealed that in vitro clonogenic expansion changed the chromosome balance (ploidy distribution) of TG180. Clonogenic expansion also resulted in a heterogeneous cell population with a smaller sub-population presenting the original near-tetraploid karyotype state (32 %) and a main sub-population (52 % of analyzed metaphases) presenting a heptaploid cytotype, with chromosome number ranging from 121 to 140 (Fig. 8g), a distribution pattern totally different of the original cell line state.

**Fig. 9:**
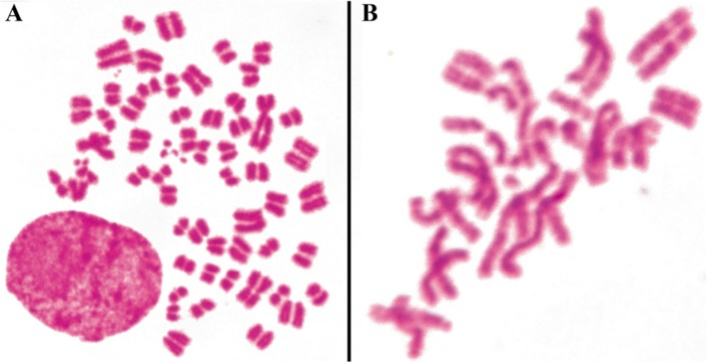
Giemsa stained metaphases of TG180. **a** and **b** TG180 metaphases showing diplochromosomes, a mark of endoreduplication occurrence in this cell line

A sample of cells derived from the clonal culture (1.0 × 10^7^ cells) was inoculated in the peritoneum of three animals and after ascetic tumor development, cells were harvested for cytogenetic analyses. Surprisingly, the original near-tetraploid main sub-population was recovered after in vivo progression. It was observed that the cells returned to their original near-tetraploid state, evidenced by 91.6 % of analyzed metaphases that presented chromosome number ranging from 61 to 80 (Fig. 8g). Clonogenic heptaploid cells probably were generated by a mechanism called endoreduplication, since diplochromosomes were observed in many metaphases of the clonal cell culture (Fig. 9a and b). This event may also have happened in the initial origin of the tumor cell line.

## Discussion

Aneuploidies characterized by complex karyotypes are the most prominent and common feature of solid tumors and tumor cell lines [24]. This chromosomal instability predisposes cells to tumor development, and has become a very intense research focus, because the mechanisms that drive tumor growth in whole chromosome aneuploidy are less well understood [25–27]. Although animal models and human cancer syndromes have been exploited to understand cancer development, very few cancer studies have used concomitant in vivo and in vitro settings to observe chromosomal behavior under different environmental conditions. We have chosen multiple in vitro and in vivo strategies, using a murine sarcoma cell line, an in vivo animal model, and in vitro cell culture, to investigate chromosomal aneuplody during tumor development and telomerase expression.

We have shown that the karyotype of the TG180 cell line kept in vitro is highly unstable, and this chromosome instability is highly associated with tumor initiation, but as tumor becomes established in the animal (in vivo condition), it tends to stabilize and select some numerical and structural chromosomal abnormalities due to telomerase up-regulation. We corroborate the notion that there is a strong link among chromosomal instability, telomere dysfunction and karyotypic variability [28], and it is possible that cells’ survival during this tumor cell line evolution might have occurred due to the increased telomerase activity, which maintains the telomere lengthening mechanism, stabilizes existing telomeres and possibly alleviates chromosome instability generated during cell cycles.

The biology and properties of cultured cells changes according to the environment of it and telomere shortening is strongly associated with chromosome instability, a signal of cancerous transformation. In their paper, Kim et al. [29], described telomere shortening during cell passages, triggering senescence after 10 passages. In some cells, they observed chromosomal aberrations at passage 5, when the telomere length was the shortest and chromosomal aberrations persisted until telomerase activity increasing. In their work telomere length gradually decreased during passaging until the point at which cytogenetic aberrations appeared. They demonstrated that rare aberrant clones at earlier passages can become predominant clones during later passages, as well as we observed when we transferred cells kept in vitro to in vivo condition.

Cellular mechanisms that sustain tumor heterogeneity is an unsolved question in cancer biology [30]. We have shown that the TG180 tumor cell line was a highly heterogeneous population of cells related to chromosome number and karyotipical in all stages of tumor development and forms of tumor maintenance. Chromosomal differences were observed in relation to in vivo and in vitro maintenance settings, suggesting that environmental changes can alter chromosomal configuration, especially when considering that animal cells (in vivo condition) were exposed to low O2 concentrations (1–10 mmHg), while in the cell culture (in vitro condition) cells was kept under high O2 concentrations (150 mmHg), which may have caused oxidative stress in cells, leading to ROS generation and impairment of antioxidant cellular defences [31], resulting in a greater chromosome instability.

Increased rate of chromosome instability in tumors generates karyotypical diversity [32], a striking feature for the maintenance of the tumor. During tumor evolution, the variable phenotypes are then subjected to clonal selection through Darwinian competition [33]. The great variability of cell phenotypes and chromosome balance observed in the present work may explain the survival and replication of tumor cells during therapies. Therefore, resistance to treatments is an adaptive response to the high selective pressure in any specific environment, where few cells with proper chromosome balance may lead to clonal propagation and proliferation.

According to Yoshioka et al. [34], cancer usually develops in conjunction with genomic instability, a characteristic observed in thecell line of this work (TG180), associated with multiple genetic mutations. Only a small number of cells, called cancer-initiating cells (CICs), are the progenitors of cancerous tissue. Genomic instability contributes to the development of CICs by directly transforming somatic stem cells, reprogramming differentiated cancer cellsfaced to different conditions found in the cellular microenvironment, and a number of other mechanisms.

Many chromosomes with four homologous containing the same chromosomal banding pattern were observed in the TG180 karyotyping, revealing for the first time the presence of tetraploidy in this cell line. Many evidences support the idea of tetraploidy as a link to aneuploidy. Several tetraploid or near-tetraploid cells have been described in the premalignant condition (Barrett’s oesophagus) [35], early-stage (cervix) [36], and even some mature cancers have near-tetraploid karyotypes [37]. Furthermore, in the present work it was detected diplochromosomes thus, it is reasonable to suggest that the standard karyotype of TG180 could be a result of an initial tetraploidy via endoreduplication, an event that drives tumor cells to acquire higher chromosome numbers [38]. Endoreduplication occurs when the cells pass through two rounds of DNA replication without chromatid separation. In this case chromatin is re-licensed even if complete mitosis does not occur [39].

In TG180, the endoreduplication probably occurred before the formation of metacentric chromosomes, because they have been observed strictly as single copy. These three metacentric chromosomes and four microchromosomes, considered as marker chromosomes, were frequently observed in cytogenetic analysis. Probably, they provide some beneficial effects and adaptive characteristics to the tumor cell line growth, thus explaining its maintenance. In this context, we suggest that these metacentric chromosomes are probably derived from Robertsonian translocation between two acrocentric chromosomes, generating micro-chromosomes as a result of arm breaks. It is reasonable to associate the origin of metacentric and microchromosomes as a result of double-stranded breaks (DSB) caused by free radicals present in tumor site during an inflammation process or due to telomeric erosion. Erroneous rejoining of broken DNA DSBs may occur, resulting in deletion or amplification of chromosome material and even translocations [40, 41]. These genetic changes are very important to tumor progression, once the resulting genomic instability can generate malignant phenotypes. In the present work, we have indirectly shown the possible influence of ROS in chromosome integrity, because cells kept in culture showed several chromosomal disorders.

The structural alterations observed in TG180 were also observed in haematological cancers [42, 43], solid tumors [44], at the onset of acute myelogenousleukemia [45], and in another murine sarcoma cell line [46]. Many chromosome translocations of tumorshave been studied and their gene fusion products identified [47]. So, it is possible that the Robertsonian translocation observed in TG180 may have caused fusion of some genes, producing chimeric proteins that may have activated cell proliferation, inactivated tumor suppressor genes or affected DNA repair [48]. Even telomerase expression may have changed as a result ofstructural chromosomal abnormalities, once that was identified by SNP-array a fusion between IRX2-TERT genes caused by an interstitial deletion in the short arm of chromosome 5 (5p15.33). The analyses revealed that IRX2 promoter dramatically upregulated TERT gene [49]. Paradoxically, an explanation for alteration in gene expression can be offered by the chromosome theory of cancer, suggesting the interdependence of these events.

Despite the presence of chromosomal rearrangements and evident aneuploidy and numerical heterogeneity, some specific chromosomes have conserved their ploidy. Whereas the third chromosome of the complement presented diploid, triploid and tetraploid forms, the first chromosome of the complement showed conserved diploidy in all analyzed metaphases, which may be a required characteristic for tumor cell survival. In order to verify this hypothetical benefit played by the conservation of some chromosomes, we carried out the NORs (nucleolus organizer regions) detection, since these regions are linked to high protein synthesis and, consequently, to the tumor aggressiveness turning interesting its conservation [50, 51]. The silver nitrate impregnation revealed increased NORs activity in all TG180 cells. In the normal cells of mouse strain used in the experiments NORs were located on chromosomes 12, 15, 16, 17, 18 and 19 [52]; however, for tumor cells, in addition to the NORs-bearing chromosomes of normal cells, we also observed activation of NORs on chromosomes 2, 4, 8, 10 and 11, coinciding with the NORs described in another mouse populations from different regions of the world [53]. Interestingly, we have observed activation of all rDNAs described for the mouse genome in the TG180 cell line, and it seemed to be positively selected during tumor development.

The conservation of specific chromosomes in TG180 karyotype led us to investigate the inheritance patterns of chromosomes in a clonogenic assay. In this experiment 50 sarcoma cells were isolated and individually cultived, of which only one showed clonogenic features and constituted a new tumor cell population. This suggests that survival and clonal proliferation is not a frequent process and only specific chromosomal balances are ideal to maintain tumor development. Although tumors are constituted by a cellular heterogeneity, only cells with the correct chromosome combinations are able to proliferate (cancer stem cells). The ideal chromosomal combination is acquired by a selection pressure after crisis state during tumor initiation, an event that causes extreme chromosomal instability, resulting in a variety of different cytotypes.

The resulting in vitro clonal expansion of a single-cell derived population showed 52 % of near-heptaploid cells (7x) and a sub-population (32 %) of near-tetraploid standard cytotype observed in the cell line (4x). Heptaploid cells were probably originated by endoreduplication during clonal expansion, since it was also observed many metaphases of TG180 with diplochromosomes in the clonal cell culture, corroborating with a study performed with a cell line derived from a primary gastric tumor [54].

Subsequently, the resulting cloned cells were inoculated in animals and, after 10 days of tumor development, cytogenetic analysis showed that most cells (91.2 %) had returned to a near-tetraploid form, as usually observed in the original cell line. Probably the near-tetraploid karyotypical arrangement presented a suitable chromosomal combination adapted to proliferate in vivo, while the heptaploid cells were only viable in cell culture, showing the importance of the microenvironment on tumor development. Once culture medium has very different conditions when compared with animal physiology [31], cells exposed to this condition are subject to different selective pressure, which directly influences its chromosome organization. Our hypothesis is that in TG180, tumor proliferative cells present near-tetraploidy formation, and only those chromosomes that are able to support in vivo growth, and changes in tumor microenvironment may influence the chromosomal architecture of these cells.

One of the most relevant characteristics of a proliferative and immortalized tumor cell is the telomerase activation or over-expression [55, 56]. A strong characteristic of very proliferative cells is the telomeric erosion that results in a crisis state [57]. Some cytogenetic studies and comparative genomic hybridization in epithelial tumors of mice with telomere dysfunction revealed a high rate of genomic aberrations among them; some non-reciprocal translocations, regional amplifications and deletions. These changes are not frequent in tumors of mice that have intact telomere function [58]. Therefore, it is clear the importance of telomerase expression to overcome crisis barrier and keep chromosomes integrity.

Although basal levels of telomerase expression are detected in mouse cells, differences can be observed in cells from distinct tissues [59]. Normal tissues with same embryonic origin of TG180, such as mesentery, peritoneal and blood cells showed very low levels of Tert mRNA, even in high proliferative cells, such as spermatogonia, which did not reach the levels of telomerase expression of TG180. Thus, telomerase overexpression possibly plays an important role in the proliferative potential of the cell line, maintaining the integrity of telomeres, and conserving the stability of the ideal chromosomal balance. High telomerase activity is correlated with fewer aberrations, ploidy regulation and high telomere signal intensity, indicating its importance to keep genoma stability [60]. We believe that genetic instability generates a crisis process in which most cells, even the ones with an ideal chromosome balance, undergo apoptosis due to telomere erosion, and just rare cells will adapt and keep telomere integrity, by telomerase over-expression, enabling proliferation and promoting tumor progression, as proposed elsewhere [61].

## Conclusions

Polyploidy via endoreduplication and aneuploidy were common events during TG180 sarcoma development and evolution, but by subjecting the cell line to a high selection pressure and to different environments, we have revealed a specific conserved chromosomal architecture, which may have promoted adaptive advantages to cancer cells. Clonal survival and expansion of tumor cells will be perpetuated by keeping the up-regulation of telomerase activity. Our data reinforce the notion that the sarcoma 180 cell evolution converges from a highly unstable karyotype to relatively stable and functional chromosome rearrangements, which are further enabled by telomerase overexpression. This is a demonstration of interdependency between chromosome and gene mutation theories for tumorigenesis, which may explain chromosome alterations, genotypic adaptation and cellular expansion.

## References

[1] Hanahan D, Weinberg RA (2011). Hallmarks of cancer: the next generation. Cell.

[2] Kroemer G, Pouyssegur J (2008). Tumor cell metabolism: cancer’s Achilles’ heel. Cancer Cell.

[3] Luo J, Solimini NL, Elledge SJ (2009). Principles of cancer therapy: oncogene and non-oncogene addiction. Cell.

[4] Negrini S, Gorgoulis VG, Halazonetis TD (2010). Genomic instability - an evolving hallmark of cancer. Nat Rev Mol Cell Biol.

[5] Beerenwinkel N, Antal T, Dingli D, Traulsen A, Kinzler KW, Velculescu VE (2007). Genetic progression and the waiting time to cancer. PLoS Comput Biol.

[6] Chen KG, Wang YC, Schaner ME, Francisco B, Durán GE, Juric D (2005). Genetic and epigenetic modeling of the origins of multidrug-resistant cells in a human sarcoma cell line. Cancer Res.

[7] Roberti A, La Sala D, Cinti C (2006). Multiple genetic and epigenetic interacting mechanisms contribute to clonally selection of drug-resistant tumors: current views and new therapeutic prospective. J Cell Physiol.

[8] Stock RP, Bialy H (2003). The sigmoidal curve of cancer. Nat Biotechnol.

[9] Davoli T, Denchi EL, De Lange T (2010). Persistent telomere damage induces bypass of mitosis and tetraploidy. Cell.

[10] Pihan G, Doxsey SJ (2003). Mutations and aneuploidy: co-conspirators in cancer?. Cancer Cell.

[11] Hanahan D, Weinberg RA (2000). The hallmarks of cancer. Cell.

[12] Bertrand V, Couturier-Turpin MH, Louvel A, Panis Y, Couturier D (1999). Relation between cytogenetic characteristics of two human colonic adenocarcinoma cell lines and their ability to grow locally or metastasize or both: an experimental study in the nude mouse. Cancer Genet Cytogenet.

[13] Chang S, Khoo C, DePinho RA (2001). Modeling chromosomal instability and epithelial carcinogenesis in the telomerase-deficient mouse. Semin Cancer Biol.

[14] Fleisig HB, Hukezalie KR, Thompson C a H, Au-Yeung TTT, Ludlow a T, Zhao CR, et al. Telomerase reverse transcriptase expression protects transformed human cells against DNA-damaging agents, and increases tolerance to chromosomal instability. Oncogene. 2015:1–10. doi:10.1038/onc.2015.75. 10.1038/onc.2015.75 25893297

[15] Chang MW, Grillaria J, Mayrhoferb C, Fortscheggera K, Allmaierb G, Marzban G (2005). Comparison of early passage, senescent and hTERT immortalized endothelial cells. Exp Cell Res.

[16] Guerra M, Souza MJ (2002). Como observar cromossomos - Um Guia de Técnicas em Citogenética Vegetal, Animal e Humana.

[17] Sumner AT (1972). A simple technique for demonstrating centromeric heterochromatin. Exp Cell Res.

[18] Verma RS, Babu A (1995). Human chromosomes: principles and techniques.

[19] Fagundes V, Vianna-Morgante AM, Yonenaga-Yassuda Y (1997). Telomeric sequences localization and G-banding patterns in the identification of a polymorphic chromosomal rearrangement in the rodent Akodon cursor (2n = 14,15 and 16). Chromosome Res.

[20] Schmid M, Almeida CG (1988). Chromosome banding in Amphibia. XII. Restriction endonuclease banding. Chromosoma.

[21] Howell WM, Black DA (1980). Controlled silver-staining of nucleolus organizer regions with a protective colloidal developer: a 1-step method. Experientia.

[22] Levan A, Fredga K, Sandberg AA (1964). Nomenclature for centromeric position on chromosomes. Hereditas.

[23] Krishan A (1975). Rapid flow cytofluorometric analysis of mammalian cell cycle by propidium iodide staining. J Cell Biol.

[24] Rondón-Lagos M, Verdun Di Cantogno L, Marchiò C, Rangel N, Payan-Gomez C, Gugliotta P (2014). Differences and homologies of chromosomal alterations within and between breast cancer cell lines: a clustering analysis. Mol Cytogenet.

[25] Duesberg P, Li R, Fabarius A, Hehlmann R (2005). The chromosomal basis of cancer. Cell Oncol.

[26] Gordon DJ, Resio B, Pellman D (2012). Causes and consequences of aneuploidy in cancer. Nat Rev Genet.

[27] Saunders WS (2000). Chromosomal instability and cytoskeletal defects in oral cancer cells. Proc Natl Acad Sci U S A.

[28] Cheung AL, Deng W (2008). Telomere dysfunction, genome instability and cancer. Front Biosci.

[29] Kim J-A, Im KO, Park SN, Kwon JS, Kim SY, Oh K (2015). Cytogenetic heterogeneity and their serial dynamic changes during acquisition of cytogenetic aberrations in cultured mesenchymal stem cells. Mutat Res.

[30] Visvader JE (2011). Cells of origin in cancer. Nature.

[31] Halliwell B (2003). Oxidative stress in cell culture: an under-appreciated problem?. FEBS Lett.

[32] Storchova Z, Pellman D (2004). From polyploidy to aneuploidy, genome instability and cancer. Nat Rev.

[33] Stephens PJ, Greenman CD, Fu B, Yang F, Bignell GR, Mudie LJ (2011). Massive genomic rearrangement acquired in a single catastrophic event during cancer development. Cell.

[34] Yoshioka K (2015). Development of cancer-initiating cells and immortalized cells with genomic instability. World J Stem Cells.

[35] Galipeau PC, Cowan DS, Sanchez CA, Barrett MT, Emond MJ, Levine DS (1996). 17p (p53) allelic losses, 4 N (G2/tetraploid) populations, and progression to aneuploidy in Barrett’s esophagus. Proc Natl Acad Sci U S A.

[36] Olaharski AJ, Sotelo R, Solorza-Luna G, Gonsebatt ME, Guzman P, Mohar A (2006). Tetraploidy and chromosomal instability are early events during cervical carcinogenesis. Carcinogenesis.

[37] Rajagopalan H, Lengauer C (2004). Aneuploidy and cancer. Nature.

[38] Larizza L, Schirrmacher V (1984). Somatic-cell fusion as a source of genetic rearrangement leading to metastatic variants. Cancer Metast Rev.

[39] Larkins BA, Dilkes BP, Dante RA, Coelho CM, Woo Y, Liu Y (2001). Investigating the hows and whys of DNA endoreduplication. J Exp Bot.

[40] Jackson SP (2002). Sensing and repairing DNA double-strand breaks. Carcinogenesis.

[41] Khanna KK, Jackson SP (2001). DNA double-strand breaks: signaling, repair and the cancer connection. Nat Genet.

[42] Qian J, Xue Y, Sun J, Guo Y, Pan J, Wu Y (2002). Constitutional Robertsonian translocations in (9;22)-positive chronic myelogenousleukemia. Cancer Genet Cytogenet.

[43] Welborn J (2004). Acquired Robertsonian translocations are not rare events in acute leukemia and lymphoma. Cancer Genet Cytogenet.

[44] Bayani J, Zielenska M, Pandita A, Al-Romaih K, Karaskova J, Harrison K (2003). Spectral karyotyping identifies recurrent complex rearrangements of chromosomes 8, 17, and 20 in osteosarcomas. Genes Chromosomes Cancer.

[45] Padilla-Nash HM, Heselmeyer-Haddad K, Wangsa D, Zhang H, Ghadimi BM, Macville M (2001). Jumping translocations are common in solid tumor cell lines and result in recurrent fusions of whole chromosome arms. Genes Chromosomes Cancer.

[46] Ghosh S, Chaudhuri A (1984). Analysis of three whole-arm translocations in a mouse sarcoma cell line. Cytogenet Cell Genet.

[47] Fletcher JA (2004). Molecular biology and cytogenetics of soft tissue sarcomas: relevance for targeted therapies. Cancer Treat Res.

[48] Rabbitts TH, Appert A, Chung G, Collins EC, Drynan L, Forster A (2001). Mouse models of human chromosomal translocations and approaches to cancer therapy. Blood Cells Mol Dis.

[49] Karlsson J, Lilljebjörn H, Holmquist Mengelbier L, Valind A, Rissler M, Øra I (2015). Activation of human telomerase reverse transcriptase through gene fusion in clear cell sarcoma of the kidney. Cancer Lett.

[50] Ishida T, Kaneko S, Akazawa K, Tateishi M, Sugio K, Sugimachi K (1993). Proliferating cell nuclear antigen expression and argyrophilicnucleolar organizer regions as factors influencing prognosis of surgically treated lung-cancer patients. Cancer Res.

[51] Kaneko S, Ishida T, Sugio K, Yokoyama H, Sugimachi K (1991). Nucleolar organizer regions as a prognostic indicator for stage-I non-small-cell lung-cancer. Cancer Res.

[52] Dev VG, Tantravahi R, Miller DA, Miller OJ (1977). Nucleolus organizers in *Mus musculus* subspecies and in the RAG mouse cell line. Genetics.

[53] Suzuki H, Kurihara Y, Kanehisa T, Moriwaki K (1990). Variation in the distribution of silver-staining nucleolar organizer regions on the chromosomes of the wild mouse, *Mus musculus*. Mol Biol Evol.

[54] Lima EM, Rissino JD, Harada ML, Assumpção PP, Demachki S, Guimarães AC (2004). Conventional cytogenetic characterization of a new cell line, ACP01, established from a primary human gastric tumor. Braz J Med Biol Res.

[55] Kim NW, Piatyszek MA, Prowse KR, Harley CB, West MD, Ho PL (1994). Specific association of human telomerase activity with immortal cells and cancer. Science.

[56] Blasco MA, Lee H, Hande MP, Samper E, Lansdorp PM, DePinho RA (1997). Telomere shortening and tumor formation by mouse cells lacking telomerase RNA. Cell..

[57] Gilley D, Tanaka H, Herbert BS (2005). Telomere dysfunction in aging and cancer. Int J Biochem Cell Biol.

[58] Maser RS, DePinho RA (2002). Connecting chromosomes, crisis, and cancer. Science.

[59] Prowse KR, Greider CW (1995). Developmental and tissue-specific regulation of mouse telomerase and telomere length. Proc Natl Acad Sci U S A.

[60] Izumi H, Hara T, Oga A, Matsuda K, Sato Y, Naito K (2002). High telomerase activity correlates with the stabilities of genome and DNA ploidy in renal cell carcinoma. Neoplasia.

[61] Shay JW, Wright WE (2011). Role of telomeres and telomerase in cancer. Semin Cancer Biol.

